# Association of 5α-Reductase Inhibitor Prescription With Bladder Cancer Progression in Males in South Korea

**DOI:** 10.1001/jamanetworkopen.2023.13667

**Published:** 2023-05-16

**Authors:** Min Ho An, Min Seo Kim, Chungsoo Kim, Tae Il Noh, Kwan Joong Joo, Dong Hun Lee, Kyu-Ho Yi, Jeong Woo Kwak, Tae-Ho Hwang, Rae Woong Park, Seok Ho Kang

**Affiliations:** 1 Department of Biomedical Informatics, Ajou University School of Medicine, Suwon, Korea; 2 Department of Medical Sciences, Graduate School of Ajou University, Suwon, Korea; 3 Samsung Advanced Institute for Health Sciences and Technology (SAIHST), Sungkyunkwan University, Samsung Medical Center, Seoul, Korea; 4 Department of Biomedical Sciences, Ajou University Graduate School of Medicine, Suwon, Korea; 5 Department of Urology, Anam Hospital, Korea University College of Medicine, Seoul, Korea; 6 Department of Urology, Soonchunhyang University Hospital, Soonchunhyang University Medical College, Seoul, Korea; 7 Department of Medicine, Ajou University College of Medicine, Suwon, Korea; 8 Division in Anatomy and Developmental Biology, Department of Oral Biology, Human Identification Research Institute, BK21 FOUR Project, Yonsei University College of Dentistry, Seoul, Korea; 9 Gyodong Health Subcenter, Incheon, Korea; 10 Department of Pharmacology, Pusan National University, School of Medicine, Yangsan, Korea; 11 Gene and Cell Therapy Research Center for Vessel-Associated Diseases, School of Medicine, Pusan National University, Yangsan, Korea

## Abstract

**Importance:**

The antiandrogenic effect of the 5α-reductase inhibitor (5-ARI) has been investigated for its role in preventing male-predominant cancers. Although 5-ARI has been widely associated with prostate cancer, its association with urothelial bladder cancer (BC), another cancer experienced predominantly by males, has been less explored.

**Objective:**

To assess the association between 5-ARI prescription prior to BC diagnosis and reduced risk of BC progression.

**Design, Setting, and Participants:**

This cohort study analyzed patient claims data from the Korean National Health Insurance Service database. The nationwide cohort included all male patients with BC diagnosis in this database from January 1, 2008, to December 31, 2019. Propensity score matching was conducted to balance the covariates between 2 treatment groups: α-blocker only group and 5-ARI plus α-blocker group. Data were analyzed from April 2021 to March 2023.

**Exposure:**

Newly dispensed prescriptions of 5-ARIs at least 12 months prior to cohort entry (BC diagnosis), with a minimum of 2 prescriptions filled.

**Main Outcomes and Measures:**

The primary outcomes were the risks of bladder instillation and radical cystectomy, and the secondary outcome was all-cause mortality. To compare the risk of outcomes, the hazard ratio (HR) was estimated using a Cox proportional hazards regression model and difference in restricted mean survival time analysis.

**Results:**

The study cohort initially included 22 845 males with BC. After propensity score matching, 5300 patients each were assigned to the α-blocker only group (mean [SD] age, 68.3 [8.8] years) and 5-ARI plus α-blocker group (mean [SD] age, 67.8 [8.6] years). Compared with the α-blocker only group, the 5-ARI plus α-blocker group had a lower risk of mortality (adjusted HR [AHR], 0.83; 95% CI, 0.75-0.91), bladder instillation (crude HR, 0.84; 95% CI, 0.77-0.92), and radical cystectomy (AHR, 0.74; 95% CI, 0.62-0.88). The differences in restricted mean survival time were 92.6 (95% CI, 25.7-159.4) days for all-cause mortality, 88.1 (95% CI, 25.2-150.9) days for bladder instillation, and 68.0 (95% CI, 31.6-104.3) days for radical cystectomy. The incidence rates per 1000 person-years were 85.59 (95% CI, 80.53-90.88) for bladder instillation and 19.57 (95% CI, 17.41-21.91) for radical cystectomy in the α-blocker only group and 66.43 (95% CI, 62.22-70.84) for bladder instillation and 13.56 (95% CI, 11.86-15.45) for radical cystectomy in the 5-ARI plus α-blocker group.

**Conclusions and relevance:**

Results of this study suggest an association between prediagnostic prescription of 5-ARI and reduced risk of BC progression.

## Key Points

**Question:**

Is prescribing a 5α-reductase inhibitor (5-ARI) prior to bladder cancer (BC) diagnosis associated with reduced risk of BC progression?

**Findings:**

In this cohort study of 22 845 males with BC, patients prescribed a 5-ARI compared with those prescribed an α-blocker had a lower risk of mortality and BC progression.

**Meaning:**

Findings of this study suggest that 5-ARIs may have protective benefits against BC progression.

## Teaser

This cohort study examines the risk of all-cause mortality, bladder instillation, and radical cystectomy in older males with filled prescriptions for a 5α-reductase inhibitor 1 year before a bladder cancer (BC) diagnosis.

## Introduction

A 5α-reductase inhibitor (5-ARI) is widely used to treat lower urinary tract symptoms in males with benign prostatic hyperplasia (BPH).^1^ Due to its antiandrogenic effects, 5-ARI has been investigated for its role in preventing male-predominant cancers, such as prostate cancer and bladder cancer (BC).^2,3^ The large Prostate Cancer Prevention Trial (PCPT)^2^ initially showed the prostate cancer–protective properties of 5-ARI, although high-grade prostate cancer was paradoxically higher in those receiving 5-ARI. This finding was validated by those of other cohort studies.^4,5,6,7,8^ In contrast, the association between 5-ARI and urothelial BC, another cancer experienced predominantly by males, remains unknown.

Urothelial BC is the sixth most prevalent cancer in males worldwide.^9^ Approximately 75% of BC cases are non–muscle-invasive bladder cancer (NMIBC) and treatable with transurethral resection of the bladder, often combined with intravesical instillation.^10^ Although survival is favorable, NMIBC progresses to muscle-invasive bladder cancer (MIBC) in up to 21% of high-risk patients.^11^ Therefore, it is important to search for pharmacotherapy to prevent muscle invasion that can be used complementarily with intravesical instillation.

To assess the association between a 5-ARI prescription prior to BC diagnosis and reduced risk of BC progression, we collected information from the Korean National Health Insurance Service (NHIS) on 22 845 patients with BC who had BPH at baseline and filled a prescription for either an α-blocker or a 5-ARI plus α-blocker. We performed propensity score matching (PSM) to offset the biases of covariates between treatment groups.^12^

## Methods

### Data Sources and Study Population

This cohort study used patient data collected by the NHIS, a governmental institution that provides health insurance coverage for approximately 97% of the population in South Korea. The NHIS data include medical eligibility (eg, income level), demographic characteristics (eg, age and sex), National Health Examination results (eg, body mass index [BMI] and smoking status), and medical treatment information (eg, date of visit, prescription name, duration, dose, and diagnosis according to the *International Statistical Classification of Diseases and Related Health Problems, Tenth Revision* [*ICD-10*] codes). The NHIS database is valid and well-suited for large population-based studies and shares similar features with the national health insurance claims databases of Taiwan and Sweden.^13,14^ The study protocol for this research was approved by the institutional review board at Korea University, and the need to obtain informed consent from the study participants was waived. This decision was based on the determination that the study was retrospective and posed minimal risk or harm to the individuals under investigation and that the potential benefits of the study surpassed any potential risks. We followed the Strengthening the Reporting of Observational Studies in Epidemiology (STROBE) reporting guideline.^15^

**Figure 1.**
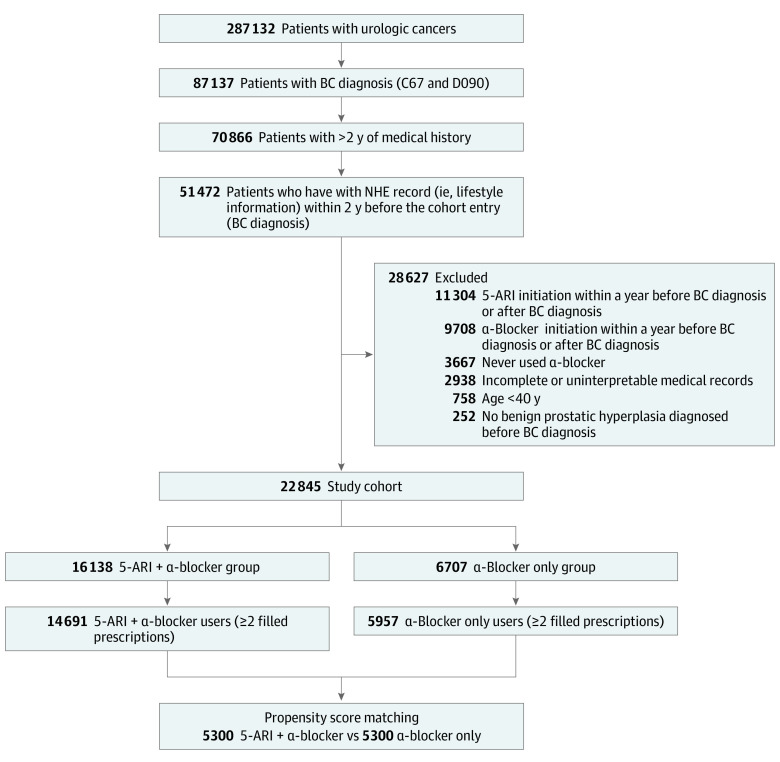
Study Flowchart and Design 5-ARI indicates 5α-reductase inhibitor; BC, bladder cancer; NHE, National Health Examination.

We identified all patients with BC from January 1, 2008, to December 31, 2019, using the NHIS database. All patients were Korean. For this study cohort, we included patients with at least 2 years of medical history in the NHIS before BC diagnosis. The entry date of the study population was the date of the first BC diagnosis. We then identified patients with National Health Examination records (eg, BMI and smoking status) within 2 years before the cohort entry date to retain patients’ life behavioral information. Patients with missing data on any variables were treated as having an incomplete record and excluded. Individuals younger than 40 years were also not included in the study. Furthermore, we structured the study cohort (Figure 1) by excluding patients who never received a prescription for an α-blocker or initiated a 5-ARI or α-blocker within a 1-year lag period prior to BC diagnosis to ensure a sufficient time window for patients who were exposed to the study drug.^4^ The lag period was defined based on the assumption that a 5-ARI may not immediately affect BC, and any BC that was diagnosed immediately after a 5-ARI prescription was likely due to factors other than drugs. This lag period was necessary not only to account for such latency but also to minimize reverse causality, whereby lower urinary symptoms associated with a yet undiagnosed or an imminently diagnosed BC warranted 5-ARI use.^4,6^ Determining the exposure status of males who had had 2 or more filled prescriptions for a 5-ARI also reduced any biases from immediate discontinuation of the medication.^8^

### Exposure, Outcomes, and Covariates

Prediagnostic prescription of a 5-ARI was defined as at least 2 filled prescriptions of a 5-ARI at any time before cohort entry (BC diagnosis), excluding prescriptions initiated in the 12 months immediately before cohort entry. The prediagnostic cumulative duration of a 5-ARI was calculated by summing the daily supply of all of the 5-ARI prescriptions that patients had filled before the initial BC diagnosis. We used an α-blocker as an active comparator to avoid confounding by indication,^16^ to improve statistical analysis (ie, PSM) by increasing the overlap of measured characteristics,^16^ and to balance the treatment effect of an α-blocker in both groups because a 5-ARI is more often prescribed secondary to an α-blocker to manage BPH in South Korea.^16,17^ An α-blocker was deemed to be a proper active comparator because it is not associated with BC risk and progression.^18^ We did not investigate the group using only 5-ARIs because the group was a small proportion of the cohort and may have received a 5-ARI prescription for alopecia. The following α-blockers were included: doxazocin (1 mg, 2 mg, and 4 mg), terazosin (1 mg, 2 mg, and 5 mg), tamsulosin (0.1 mg, 0.2 mg, and 0.4 mg), alfuzosin (10 mg), silodosin (4 mg and 8 mg), and naftopidil (25 mg, 50 mg, and 75 mg). Prediagnostic prescription of an α-blocker was defined as at least 2 filled prescriptions of an α-blocker at any time before cohort entry (BC diagnosis), excluding prescriptions initiated in the 12 months immediately before cohort entry.

We identified all patients with BC who had *ICD-10* codes D09.0 (carcinoma in situ of bladder) or C67.x (malignant neoplasm of bladder), whichever was diagnosed first. The primary outcomes were the risks of bladder instillation and radical cystectomy, and the secondary outcome was all-cause mortality. For each outcome, patients were followed up from BC diagnosis to the event of interest or the last follow-up in the cohort, whichever occurred first.

We extracted comorbidities to calculate the Charlson Comorbidity Index (CCI)^19^ based on patients’ medical history (eTable 1 in Supplement 1) of myocardial infarction, congestive heart failure, peripheral vascular disease, cerebrovascular disease, dementia, chronic pulmonary disease, rheumatologic disease, peptic ulcer disease, mild liver disease, diabetes without chronic complication, diabetes with chronic complications, hemiplegia or paraplegia, kidney disease, any malignant neoplasm (including leukemia and lymphoma), moderate or severe liver disease, metastatic solid tumor, and AIDS or HIV. The CCI (categorized into 0, 1-2, ≥3, with the higher scores indicating more severe comorbidity) was calculated for each patient at cohort entry. The calendar year at BC diagnosis and other cancer diagnoses prior to BC diagnosis was also curated. Moreover, other BPH medications, such as β3-agonists and anticholinergics (prescribed at any time until the 12 months before the cohort entry), in addition to nonurologic medications (antihypertensive, antiatherosclerotic, and hypoglycemic agents and male hormone) were collected (measured at baseline within 1 year from cohort entry). Individuals with missing data on exposures, outcomes, and/or covariates were excluded from the study without imputation.

### Statistical Analysis

Descriptive statistics were used to summarize the characteristics of each drug group. Propensity score matching was performed with PSMATCH in SAS (SAS Institute Inc). Propensity scores were calculated using the greedy nearest neighbor matching method and a caliper value of 0.02. After matching, all covariates were well-balanced between the treatment groups with a standardized mean difference of less than 0.10.^20^ Compared with other propensity score methods such as propensity score stratification and inverse probability of treatment weighting that compromise covariate balance to retain all study samples, PSM ensures excellent covariate balance at the expense of sacrificing sample size by stringently excluding unmatched patients.^21,22^ We prioritized covariate balance because even minor differences in covariates between treatment groups may yield false-positive signals that could be incorrectly attributed to drugs, and intensive exclusion of the unmatched population was tolerable with the data set of this large sample size. We tested the proportional hazards assumption with Schoenfeld residuals technique,^23^ and we further inspected the 0 slope in Schoenfeld residual plot and crossover in Kaplan-Meier plot.^24^ The cumulative risks between the 2 groups were plotted with a Kaplan-Meier cumulative risk function for the outcomes that did not violate proportionality assumption, and a log-rank test was performed to compare treatment groups. Cox proportional hazards regression models were used to estimate hazard ratios (HRs) and 95% CIs; the models were further adjusted for demographic factors, including age, smoking status, BMI, CCI, and income level. Restricted mean survival time (RMST) was provided if nonproportionality was evident.^25^ The RMST is the mean length of time (days) that patients remain free of an event until a certain point in time.^25,26^ It is calculated by measuring the area under the Kaplan-Meier curve from the beginning of the study until the prespecified time point, and it is not dependent on proportional hazards assumption.^25^ We ran RMST analyses, truncating at the time of the last follow-up.^27^ Crude incidence rates with 95% CIs were calculated by dividing the number of patients with a given outcome by the person-time at risk, based on a Poisson distribution.^4^

We replicated the analysis exclusively in patients with continuous use (eTable 2 in Supplement 1) because intermittent drug use with substantial on and off prescription could raise biases. Continuous use of a 5-ARI is defined as a maximum prescription interval not longer than 30 days. To prevent biased results associated with short-term drug use, we also conducted sensitivity analyses by excluding individuals with a cumulative duration of all 5-ARI prescriptions of less than 180 days and less than 365 days. The duration-response association with BC progression (bladder instillation and radical cystectomy) was assessed in the most conservative populations (≥365 days of filled prescriptions). Cox proportional hazards regression models were used to estimate adjusted HRs (AHRs) for each duration subgroup using the α-blocker only group as the reference. Given the uncertainties related to the choice of the 12-month exposure window prior to BC diagnosis, we performed a sensitivity analysis by varying the lengths of the time window (12, 18, and 24 months).

Two-sided *P* < .05 indicated statistical significance. All analyses were performed using SAS Enterprise Guide, version 7.1 (SAS Institute Inc), and R, version 4.0.3 (R Foundation for Statistical Computing). Data were analyzed from April 2021 to March 2023.

## Results

From 2008 to 2019, 287 132 patients with urological cancer were enrolled, of whom 87 137 were diagnosed with BC (Figure 1). Among these, 51 472 patients had at least 2 years of previous medical history and National Health Examination records (eg, lifestyle factors such as drinking alcohol and smoking cigarettes) before the cohort entry date (BC diagnosis). A total of 28 627 patients were excluded due to incomplete or uninterpretable medical records, absence of BPH diagnosis, or a short interval between the initiation of a 5-ARI or an α-blocker and BC diagnosis. Those who filled the prescription for drugs only once were excluded, and the final cohort consisted of 22 845 males who were matched using propensity scores. A total of 5300 matched patients were assigned to each treatment group: α-blocker only group (mean [SD] age, 68.3 [8.8] years) and 5-ARI plus α-blocker group (mean [SD] age, 67.8 [8.6] years).

Before PSM, the 5-ARI plus α-blocker group compared with the α-blocker only group had a higher mean (SD) age (71.7 [8.3] years vs 66.6 [9.8] years), mean (SD) duration of an α-blocker prescription (1221.2 [993.4] days vs 618.4 [808.7] days), percentage of anticholinergic prescription (37.4% vs 26.1%), percentage of antihypertensive prescription (57.7% vs 51.6%), and higher values in several other covariates (Table 1; eFigure 1 in Supplement 1). After PSM, the 2 treatment groups were comparable in all covariates (standardized mean difference <0.10) (Table 1). The propensity score distribution between treatment groups after matching was comparable (eFigure 2 in Supplement 1). eFigure 3 in Supplement 1 shows how the study cohort was constructed. The mean (SD) follow-up period from study cohort entry to death or last follow-up was 1203.1 (863.2) days for the 5-ARI plus α-blocker group and 1120.9 (839.3) days for the α-blocker only group. The proportional hazards assumption (Schoenfeld residual test) was violated for all-cause mortality and bladder instillation in patients with all use but not in patients with continuous use (eFigure 4 in Supplement 1). Crossover of Kaplan-Meier curves in all-cause mortality and bladder instillation indicated evidence of nonproportionality (eFigure 5 in Supplement 1). Therefore, we provided RMST analysis results in parallel.

**Table 1.** Baseline Characteristics of Patients Before and After PSM

Characteristic	Before PSM, No. (%)		After PSM, No. (%)		SMD^a^
	α-Blocker only group	5-ARI (+ α-blocker) group	α-Blocker only group	5-ARI (+ α-blocker) group	
No. of patients	5957	14 691	5300	5300	
Age, mean (SD), y	66.6 (9.8)	71.7 (8.3)	68.3 (8.8)	67.8 (8.6)	0.05
Smoking status					
Nonsmoker	1997 (33.5)	5807 (39.5)	1862 (35.1)	1870 (35.3)	0.01
Previous smoker	2592 (43.5)	6346 (43.2)	2315 (43.7)	2282 (43.1)	
Current smoker	1368 (23.0)	2538 (17.3)	1123 (21.2)	1148 (21.7)	
BMI, mean (SD)	24.1 (3.0)	24.1 (3.0)	24.1 (3.0)	24.1 (2.9)	0.003
α-Blocker prescription duration before BC diagnosis, mean (SD), d	618.4 (808.7)	1221.2 (993.4)	680.5 (835.3)	741.6 (772.0)	0.08
5-ARI prescription duration until the last follow-up, mean (SD), d	NA	1061.9 (1016.7)	NA	786.4 (885.0)	NA
5-ARI prescription duration until the BC diagnosis, mean (SD), d	NA	717.6 (772.8)	NA	468.4 (584.8)	NA
CCI, mean (SD)^b^	4.7 (2.5)	5.1 (2.5)	4.8 (2.5)	4.8 (2.5)	0.003
CCI group					
0	142 (2.4)	236 (1.6)	119 (2.2)	108 (2.0)	0.02
1-2	1059 (17.8)	2005 (13.6)	855 (16.1)	838 (15.8)	
≥3	4756 (79.8)	12450 (84.7)	4326 (81.6)	4354 (82.2)	
Income level					
Low	1231 (20.7)	3192 (21.7)	1149 (21.7)	1101 (20.8)	0.03
Middle	1499 (25.2)	3489 (23.7)	1340 (25.3)	1310 (24.7)	
High	3227 (54.2)	8010 (54.5)	2811 (53.0)	2889 (54.5)	
BC diagnosis year					
2010-2013	1327 (22.3)	3069 (20.9)	1184 (22.3)	1314 (24.8)	0.07
2014-2016	2131 (35.8)	5262 (35.8)	1868 (35.2)	1911 (36.1)	
2017-2019	2499 (42.0)	6360 (43.3)	2248 (42.4)	2075 (39.2)	
Other cancers before BC diagnosis	3332 (55.9)	8764 (59.7)	3041 (57.4)	2953 (55.7)	0.03
α-Blocker	5957 (100.0)	14 691 (100.0)	5300 (100.0)	5300 (100.0)	<0.001
β-3 Agonist	278 (4.7)	1026 (7.0)	262 (4.9)	256 (4.8)	0.005
Anticholinergics	1556 (26.1)	5498 (37.4)	1480 (27.9)	1470 (27.7)	0.004
Antihypertensive agent	3072 (51.6)	8472 (57.7)	2871 (54.2)	2812 (53.1)	0.02
Antiatherosclerotic drug	2346 (39.4)	6173 (42.0)	2169 (40.9)	2130 (40.2)	0.02
Male hormone	11 (0.2)	16 (0.1)	9 (0.2)	9 (0.2)	<0.001
Hypoglycemic agent	1335 (22.4)	3447 (23.5)	1226 (23.1)	1213 (22.9)	0.006

Abbreviations: 5-ARI, 5α-reductase inhibitor; BC, bladder cancer; BMI, body mass index (calculated as weight in kilograms divided by height in meters squared); CCI, Charlson Comorbidity Index; NA, not applicable; PSM, propensity score matching; SMD, standardized mean difference.

^a^ SMD greater than 0.10 can be considered a sign of imbalance.

^b^ To score the CCI, patients’ previous history of myocardial infarction, congestive heart failure, peripheral vascular disease, cerebrovascular disease, dementia, chronic pulmonary disease, rheumatologic disease, peptic ulcer disease, mild liver disease, diabetes without chronic complication, diabetes with chronic complication, hemiplegia or paraplegia, kidney disease, any malignant neoplasm (including leukemia and lymphoma), moderate or severe liver disease, metastatic solid tumor, and AIDS or HIV were collected.

### Primary Outcomes

Prescription of a 5-ARI was associated with a lower risk of bladder instillation (crude HR, 0.84; 95% CI, 0.77-0.92). The result was consistent after adjusting for covariates in the primary analysis (AHR, 0.84; 95% CI, 0.77-0.92) and in sensitivity analyses for individuals whose prescriptions were filled for 180 days or more (AHR, 0.82; 95% CI, 0.73-0.93) or 365 days or more (AHR, 0.85; 95% CI, 0.74-0.97) (Table 2). The RMST for bladder instillation was significantly longer with the 5-ARI plus α-blocker group (RMST difference, 88.1 [95% CI, 25.2-150.9] days) (Table 2) overall. The Kaplan-Meier curves for bladder instillation diverged early on; however, the early advantage of treatment was not sustained, and the cumulative incidence curves converged later (eFigure 5 in Supplement 1). These results may indicate diminishing treatment effect.^25^ The incidence rates of bladder instillation were 85.59 (95% CI, 80.53-90.88) per 1000 person-years in the α-blocker only group and 66.43 (95% CI, 62.22-70.84) per 1000 person-years in the 5-ARI plus α-blocker group (eTable 3 in Supplement 1).

**Table 2.** Primary and Sensitivity Analyses of the Association Between 5-ARI Prescriptions and Progression in Patients With Bladder Cancer

	Proportional HR^a^		RMST	
	Crude (95% CI)	Adjusted (95% CI)^b^	Difference in RMST (95% CI), d	*P* value
**Bladder instillation**				
Primary analysis				
≥2 Filled prescriptions of 5-ARIs	0.84 (0.77 to 0.91)	0.84 (0.77 to 0.92)	88.1 (25.2 to 150.9)	.006
Sensitivity analysis				
≥180 d Filled prescriptions	0.82 (0.73 to 0.92)	0.82 (0.73 to 0.93)	98.8 (25.1 to 172.5)	.009
≥365 d Filled prescriptions	0.85 (0.74 to 0.98)	0.85 (0.74 to 0.97)	64.1 (−23.7 to 151.8)	.15
**Radical cystectomy**				
Primary analysis				
≥2 Filled prescriptions of 5-ARIs	0.73 (0.62 to 0.87)	0.74 (0.62 to 0.88)	68.0 (31.6 to 104.3)	<.001
Sensitivity analysis				
≥180 d Filled prescriptions	0.69 (0.54 to 0.82)	0.70 (0.55 to 0.89)	67.6 (23.8 to 111.4)	.003
≥365 d Filled prescriptions	0.73 (0.56 to 0.95)	0.72 (0.55 to 0.94)	53.4 (4.7 to 102.1)	.03

Abbreviations: 5-ARI, 5α-reductase inhibitor; HR, hazard ratio; RMST, restricted mean survival time.

^a^ Cox proportional hazards regression model in the matched cohort.

^b^ The models were adjusted for age, smoking status, body mass index, Charlson Comorbidity Index (CCI), and income level. To score the CCI, patients’ previous history of myocardial infarction, congestive heart failure, peripheral vascular disease, cerebrovascular disease, dementia, chronic pulmonary disease, rheumatologic disease, peptic ulcer disease, mild liver disease, diabetes without chronic complication, diabetes with chronic complication, hemiplegia or paraplegia, kidney disease, any malignant neoplasm (including leukemia and lymphoma), moderate or severe liver disease, metastatic solid tumor, and AIDS or HIV were collected.

**Figure 2.**
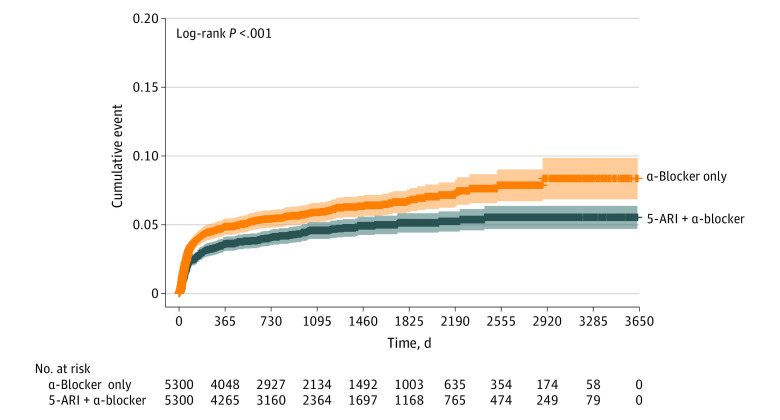
Kaplan-Meier Cumulative Risk Curve for Radical Cystectomy The shaded areas represent the 95% CIs.

The risk of radical cystectomy was lower in the 5-ARI plus α-blocker group (log-rank *P* < .001) (Figure 2), which was consistent after adjusting for covariates in the primary analysis (AHR, 0.74; 95% CI, 0.62-0.88) and sensitivity analyses for individuals whose prescriptions were filled for 180 days or more (AHR, 0.70; 95% CI, 0.55-0.89) or 365 days or more (AHR, 0.72; 95% CI, 0.55-0.94) (Table 2). The RMST for radical cystectomy was significantly longer with the 5-ARI plus α-blocker group (RMST difference, 68.0 [95% CI, 31.6-104.3] days), and the results were consistent in sensitivity analyses (Table 2). Compared with an α-blocker prescription with corresponding durations, only a 5-ARI duration for longer than 2 years (730 days) was associated with a reduced risk of radical cystectomy (AHR, 0.62; 95% CI, 0.42-0.91) (Table 3). The incidence rates of radical cystectomy were 19.57 (95% CI, 17.41-21.91) per 1000 person-years in the α-blocker only group and 13.56 (95% CI, 11.86-15.45) per 1000 person-years in the 5-ARI plus α-blocker group (eTable 3 in Supplement 1).

**Table 3.** Association of Cumulative Prediagnostic Duration of 5-ARI Prescription With Radical Cystectomy in Patients With a Filled Prescription of 5-ARI for ≥1 Year^a^

Prediagnostic prescription duration of 5-ARI	Group	No.		HR (95% CI)	
		Exposed	Cases	Crude	Adjusted
1-2 y	α-Blocker only	788	53	1 [Reference]	1 [Reference]
1-2 y	5-ARI + α-blocker	1137	58	0.74 (0.51-1.07)	0.74 (0.51-1.08)
≥2 y	α-Blocker only	1699	75	1 [Reference]	1 [Reference]
≥2 y	5-ARI + α-blocker	1350	38	0.62 (0.42-0.92)	0.62 (0.42-0.91)

Abbreviations: 5-ARI, 5α-reductase inhibitor; HR, hazard ratio.

^a^ Cumulative duration of 5-ARI was determined by adding the daily supply of all of the 5-ARI prescriptions patients had before being diagnosed with bladder cancer. Models were adjusted for age, smoking status, body mass index, Charlson Comorbidity Index, and income level.

### Secondary Outcomes

A significantly lower risk of all-cause death was observed in patients who filled a 5-ARI plus α-blocker prescription (crude HR, 0.82; 95% CI, 0.75-0.90), which was consistent after adjusting for covariates in the Cox proportional hazards regression model (AHR, 0.83; 95% CI, 0.75-0.91) (eTable 3 in Supplement 1). The RMST for death was significantly longer with the 5-ARI plus α-blocker group (RMST difference, 92.6 [95% CI, 25.7-159.4] days) (eTable 3 in Supplement 1). The incidence rates of all-cause death were 46.08 (95% CI, 42.95-49.38) per 1000 person-years in the 5-ARI plus α-blocker group and 56.53 (95% CI, 52.93-60.30) per 1000 person-years in the α-blocker only group (eTable 3 in Supplement 1).

### Sensitivity Analysis

The results for primary and secondary outcomes were consistent in a sensitivity analysis for patients with continuous use of a 5-ARI (eTable 4 and eFigure 5 in Supplement 1). Associations of 5-ARI prescription with radical cystectomy were significant only when a 5-ARI was prescribed for 2 years or more in both cumulative duration (AHR, 0.62; 95% CI, 0.42-0.98) and total duration (AHR, 0.77; 95% CI, 0.62-0.95) analyses (Table 3; eTable 5 in Supplement 1). Moreover, the results did not differ by diverse exposure lag periods prior to BC diagnosis (eTable 6 in Supplement 1). The difference in RMST for bladder instillation turned into the null when excluding people who had not filled a prescription for longer than 365 days (RMST difference, 64.1 [95% CI, –23.7 to 151.8] days) (Table 2). In a sensitivity analysis with 365 days or more filled prescriptions that used diverse truncated time point for RMST analysis, the RMST differences were significant when truncated time was 8 years or less (eTable 7 in Supplement 1).

## Discussion

To our knowledge, this cohort study was the first nationwide and matched analysis to determine the association between a 5-ARI prescription and BC progression. We found that prescribing a 5-ARI before BC diagnosis was associated with reduced risks of all-cause mortality, intravesical instillation, and radical cystectomy. Less frequent intravesical instillation after a 5-ARI prescription suggests that this drug is associated with delayed progression to a higher grade of NMIBC. Reduced radical cystectomy with a 5-ARI suggests that this medication has a role in prevention of muscle invasion. This hindrance of BC progression partially explains the decrease in all-cause mortality associated with a 5-ARI prescription.

We performed PSM to create a target population whose covariates were balanced across diverse baseline characteristics (Table 1).^12^ Investigations of nonindicated drug reactions or drug safety issues (pharmacovigilance) could be vulnerable to confounders because drug-related adverse events occur rarely and often long after drug exposure.^28^ Therefore, minor differences in covariates between groups may yield false-positive signals that could be incorrectly attributed to drugs. Even a randomized clinical trial (RCT), the gold standard design, is less informative for evaluating rare events or long-term adverse outcomes associated with drugs.^29^ Propensity score matching is a validated strategy and widely used in pharmacovigilance studies to reduce bias and mimic long-term RCTs.^12,30^ The present cohort study would benefit from an unprecedented covariate balance because, to our knowledge, no previous study has used matching methods when exploring the association between 5-ARIs and urological cancers.^3,4,5,6,7,8,18^

The findings in this study align with those of a previous study by Mäkelä et al,^18^ who observed lower BC death (HR, 0.84; 95% CI, 0.73-0.97) in patients who filled a 5-ARI prescription than in those who did not use the drug. Mäkelä et al^18^ also observed fewer cystectomies (6.8% vs 9.4%) and chemotherapies (20.1% vs 22.8%) in patients with a 5-ARI prescription compared with those without. However, the analysis was conducted in an unmatched cohort and did not account for smoking behavior, one of the strongest risk factors for BC.^31^ Another notable difference between the present study and the study by Mäkelä et al^18^ was that those authors analyzed Finnish males, whereas we focused on an Asian population. Given that people with different ethnic backgrounds could have varied BC outcomes,^32^ the results from 2 ethnically different cohorts should be considered as ethnically specific. Shiota et al^33^ expanded the scope of a 5-ARI administration in patients with BC by showing a reduced recurrence rate of BC after androgen deprivation therapy and a 5-ARI. Nonetheless, their results should be interpreted with caution: their statistical power may be limited due to small sample size and few cases (228 males, including 32 with and 196 without androgen suppression therapy).^33^

The findings can be attributed to androgen receptor (AR)–mediated mechanisms and the cause of BC. Many in vitro and in vivo studies have been conducted to understand how BC prevalence varies by sex. Androgen receptor is highly expressed in BC tissues, and its role in bladder carcinogenesis was revealed in an AR knockout mouse model.^34,35^ Overdevest et al^36^ found that overexpression of the glycosylated mucin-like protein CD24 drives tumorigenesis and metastasis in BC in an androgen-dependent manner. Furthermore, a 5-ARI reduces the conversion of dihydrotestosterone by antagonizing 5α-reductase and inhibits the proliferation of cancer cells in the bladder by altering the synthesis of certain transcripts (ie, circular RNA) that exacerbate BC.^37,38^ Concordant experimental evidence around AR-mediated carcinogenesis and tumor progression of BC supports the potential role of a 5-ARI in prevention of BC progression in large populations.

Unlike previous preclinical attempts to explain the male predominance of BC through AR signaling, population-based data have not indicated a correlation between AR expression and sex in BC.^39,40,41^ Females with BC may therefore also experience the AR-mediated benefit of a 5-ARI; however, this benefit should be explored in future studies because there is currently no empirical evidence that supports the efficacy of 5-ARI administration in females with BC. Direct exposure of pregnant individuals to 5-ARIs has been associated with birth defects (US Food and Drug Administration pregnancy category X)^42,43^; prescribing a 5-ARI to reproductive-aged individuals, therefore, warrants extreme caution.

### Limitations

This study has limitations. First, exact BC staging information could not be retrieved due to the inherent limitations of the NHIS database. Nevertheless, lack of this information may not substantially weaken the findings because BC management practices are standardized, and the treatment modality (transurethral resection of the bladder, bladder instillation, or cystectomy) itself represents the clinical status (NMIBC vs MIBC) of BC. For instance, radical cystectomy is performed in patients with MIBC (≥stage T2) or high-risk and unresponsive NMIBC who already have undetected MIBC or will likely develop MIBC.^44^ Therefore, intravesical instillation and radical cystectomy serve as proxies for BC progression. Furthermore, BC progression was an outcome, not an exposure, of this study. Second, we could not retrieve BC-specific mortality due to limitations of the NHIS database, which we deemed to be unsuitable for determining cancer-specific mortality because biases due to outcome misclassification are prevalent (>20%) in pharmacoepidemiological studies of observational databases.^45,46^ Instead, we analyzed all-cause mortality to avoid the bias inherent in the determination of causes of death.^47^ Third, the favorable findings regarding the duration of a 5-ARI prescription should be scrutinized. The likelihood of undergoing radical cystectomy was null for those who filled a 5-ARI prescription for less than 2 years (Table 3), and a 5-ARI was effective only when prescribed for longer periods. This finding may indicate the dose-dependent protection of a 5-ARI against BC progression.

Fourth, we used filled prescription data without data on serum drug levels; thus, we cannot guarantee that prescribed medications were actually used by patients. We addressed this issue by performing sensitivity analyses that exclusively included prescriptions that were cumulatively filled for more than 180 days and 360 days (Table 2), as it was unlikely that a patient who was not consuming the drug would continue to refill the prescription.^8^ These sensitivity analyses also prevented biased results from those with short-term drug use. Fifth, in the primary analysis, we assessed cumulative drug duration, regardless of the use pattern (eg, temporarily stop prescribing and then renewing the drug). We conducted a sensitivity analysis for patients with continuous use (excluding intermittent use) to minimize on-and-off drug prescription patterns and observed consistent results. Sixth, the reduction in sample size from 22 845 was mainly due to the small number of patients in the α-blocker only group (n = 5957). The loss in sample size was not substantial (from 5957 to 5300) and less likely to raise the potential issue of generalizability or bias. Seventh, the sample included only males, and therefore the findings should not be directly extrapolated to females with BC or BC with AR-independent cause.

## Conclusions

This cohort study found that the prediagnostic prescription of a 5-ARI was associated with a reduced risk of BC progression. However, the evidence was insufficient to inform the extension of 5-ARI indications. A prospective cohort study or RCT is needed to validate the findings of this study.

## Notes

Supplement 1. **eTable 1.** Lists of Charlson's Comorbidities **eTable 2.** Baseline Characteristics for Continuous Users **eTable 3.** Crude and Adjusted Hazard Ratios of Clinical Outcomes Associated With ≥ 2 Filled Prescriptions of 5ARIs in Bladder Cancer Patients **eTable 4.** Crude and Adjusted Hazard Ratios of Clinical Outcomes Associated With Continuous User of 5ARIs in Bladder Cancer Patients **eTable 5.** The Association Between Total Pre-Diagnostic Duration of 5ARI Prescription and Radical Cystectomy in Those With 365 Days or More Filled Prescription of 5ARI **eTable 6.** The Association Between 5ARI Prescriptions (≥2 Filled) and Progression in Bladder Cancer Patients Based on Different Exposure Lag Periods Prior to Bladder Cancer Diagnosis **eTable 7.** Sensitivity Analysis for RMST Using Diverse Truncated Time Point for the Association Between 5ARI Prescriptions and Bladder Instillation (365 Days or More Filled Prescriptions) **eFigure 1.** Covariate Balance Between Arms Before and After Propensity Score Matching (PSM) for (A) All Users and (B) Continuous Users **eFigure 2.** Propensity Score Distribution of Arms After Matching for All Users (A,B) and Continuous Users (C,D) **eFigure 3.** Diagram of Cohort Construction **eFigure 4.** Diagnostics for the Proportional Hazards Assumption of the Cox Proportional Hazards Model Using Schoenfeld Residuals **eFigure 5.** Cumulative Incidence Plot of Clinical Outcomes for All (A,B,C) and Continuous 5ARI Users (D,E,F)

Supplement 2.
Data Sharing Statement
