# Supplementary material for: Association of 5α-Reductase Inhibitor Prescription With Bladder Cancer Progression in Males in South Korea
Source: JAMA Netw Open. 2023 May 16;6(5):e2313667. doi: 10.1001/jamanetworkopen.2023.13667 (PMC10189569; doi:10.1001/jamanetworkopen.2023.13667)
Supplement: Supplement 1. — eTable 1. Lists of Charlson's Comorbidities eTable 2. Baseline Characteristics for Continuous Users eTable 3. Crude and Adjusted Hazard Ratios of Clinical Outcomes Associated With ≥ 2 Filled Prescriptions of 5ARIs in Bladder Cancer Patients eTable 4. Crude and Adjusted Hazard Ratios of Clinical Outcomes Associated With Continuous User of 5ARIs in Bladder Cancer Patients eTable 5. The Association Between Total Pre-Diagnostic Duration of 5ARI Prescription and Radical Cystectomy in Those With 365 Days or More Filled Prescription of 5ARI eTable 6. The Association Between 5ARI Prescriptions (≥2 Filled) and Progression in Bladder Cancer Patients Based on Different Exposure Lag Periods Prior to Bladder Cancer Diagnosis eTable 7. Sensitivity Analysis for RMST Using Diverse Truncated Time Point for the Association Between 5ARI Prescriptions and Bladder Instillation (365 Days or More Filled Prescriptions) eFigure 1. Covariate Balance Between Arms Before and After Propensity Score Matching (PSM) for (A) All Users and (B) Continuous Users eFigure 2. Propensity Score Distribution of Arms After Matching for All Users (A,B) and Continuous Users (C,D) eFigure 3. Diagram of Cohort Construction eFigure 4. Diagnostics for the Proportional Hazards Assumption of the Cox Proportional Hazards Model Using Schoenfeld Residuals eFigure 5. Cumulative Incidence Plot of Clinical Outcomes for All (A,B,C) and Continuous 5ARI Users (D,E,F) [file jamanetwopen-e2313667-s001.pdf]

Supplemental Online Content

An MH, Kim MS, Kim C, et al. Association of 5α-reductase inhibitor prescription with bladder cancer progression in males in South Korea. *JAMA Netw Open*. 2023;6(5):e2313667. doi:10.1001/jamanetworkopen.2023.13667

eTable 1. Lists of Charlson's Comorbidities

eTable 2. Baseline Characteristics for Continuous Users

eTable 3. Crude and Adjusted Hazard Ratios of Clinical Outcomes Associated With ≥ 2 Filled Prescriptions of 5ARIs in Bladder Cancer Patients

eTable 4. Crude and Adjusted Hazard Ratios of Clinical Outcomes Associated With Continuous User of 5ARIs in Bladder Cancer Patients

eTable 5. The Association Between Total Pre-Diagnostic Duration of 5ARI Prescription and Radical Cystectomy in Those With 365 Days or More Filled Prescription of 5ARI

eTable 6. The Association Between 5ARI Prescriptions (≥2 Filled) and Progression in Bladder Cancer Patients Based on Different Exposure Lag Periods Prior to Bladder Cancer Diagnosis

eTable 7. Sensitivity Analysis for RMST Using Diverse Truncated Time Point for the Association Between 5ARI Prescriptions and Bladder Instillation (365 Days or More Filled Prescriptions)

eFigure 1. Covariate Balance Between Arms Before and After Propensity Score Matching (PSM) for (A) All Users and (B) Continuous Users

eFigure 2. Propensity Score Distribution of Arms After Matching for All Users (A,B) and Continuous Users (C,D)

eFigure 3. Diagram of Cohort Construction

eFigure 4. Diagnostics for the Proportional Hazards Assumption of the Cox Proportional Hazards Model Using Schoenfeld Residuals

eFigure 5. Cumulative Incidence Plot of Clinical Outcomes for All (A,B,C) and Continuous 5ARI Users (D,E,F)

This supplemental material has been provided by the authors to give readers additional information about their work.

© 2023 An MH et al. *JAMA Network Open*

eTable 1. Lists of Charlson's comorbidities

Comorbidities	International Classification of Disease, 10th revision code
Myocardial infarction	I21.x, I22.x, I25.2
Congestive heart failure	I09.9, I11.0, I13.0, I13.2, I25.5, I42.0, I42.5-I42.9, I43.x, I50.x, P29.0
Peripheral vascular disease	I70.x, I71.x, I73.1, I73.8, I73.9, I77.1, I79.0, I79.2, K55.1, K55.8, K55.9, Z95.8, Z95.9
Cerebrovascular disease	G45.x, G46.x, H34.0, I60.x-I69.x
Dementia	F00.x-F03.x, F05.1, G30.x, G31.1
Chronic pulmonary disease	I27.8, I27.9, J40.x-J47.x, J60.x-J67.x, J68.4, J70.1, J70.3
Rheumatologic disease	M05.x, M06.x, M31.5, M32.x-M34.x, M35.1, M35.3, M36.0
Peptic ulcer disease	K25.x-K28.x
Mild liver disease	B18.x, K70.0-K70.3, K70.9, K71.3-K71.5, K71.7, K73.x, K74.x, K76.0, K76.2-K76.4, K76.8, K76.9, Z94.4
Diabetes without chronic complication	E10.0, E10.1, E10.6, E10.8, E10.9, E11.0, E11.1, E11.6, E11.8, E11.9, E12.0, E12.1, E12.6, E12.8, E12.9, E13.0, E13.1, E13.6, E13.8, E13.9, E14.0, E14.1, E14.6, E14.8, E14.9
Diabetes with chronic complication	E10.2-E10.5, E10.7, E11.2-E11.5, E11.7, E12.2-E12.5, E12.7, E13.2-E13.5, E13.7, E14.2-E14.5, E14.7
Hemiplegia or paraplegia	G04.1, G11.4, G80.1, G80.2, G81.x, G82.x, G83.0-G83.4, G83.9
Renal disease	I12.0, I13.1, N03.2-N03.7, N05.2-N05.7, N18.x, N19.x, N25.0, Z49.0-Z49.2, Z94.0, Z99.2
Any malignancy including leukemia and lymphoma	C00.x-C26.x, C30.x-C34.x, C37.x-C41.x, C43.x, C45.x-C58.x, C60.x-C76.x, C81.x-C85.x, C88.x, C90.x-C97.x
Moderate or severe liver disease	I85.0, I85.9, I86.4, I98.2, K70.4, K71.1, K72.1, K72.9, K76.5, K76.6, K76.7
Metastatic solid tumor	C77.x-C80.x
Acquired immune deficiency syndrome/ human immunodeficiency virus	B20.x-B22.x, B24.x

© 2023 An MH et al. *JAMA Network Open*

eTable 2. Baseline characteristics for continuous users

	Before propensity score matching		After propensity score matching		
	AB only	5ARI (+AB)	AB only	5ARI (+AB)	SMD
Number of patients	5957	4361	3647	3647	
Age, mean (SD)	66.57 (9.79)	71.09 (8.41)	70.35 (8.42)	69.92 (8.24)	0.051
Smoking, n (%)					0.012
Non-smoker	1997 (33.5)	1626 (37.3)	1338 (36.7)	1326 (36.4)	
Previous smoker	2592 (43.5)	1977 (45.3)	1638 (44.9)	1634 (44.8)	
Current smoker	1368 (23.0)	758 (17.4)	671 (18.4)	687 (18.8)	
Body mass index (BMI), mean (SD)	24.09 (2.95)	24.04 (2.93)	24.05 (2.94)	24.05 (2.95)	0.001
Alpha blockers prescription duration before BC diagnosis, mean (SD), days	618.42 (808.64)	1104.90 (997.14)	855.95 (925.91)	906.65 (881.17)	0.056
5ARI prescription duration until the end of follow-up, mean (SD), days	NaN (NA)	971.25 (1092.96)	NaN (NA)	840.75 (1010.03)	NA
5ARI prescription duration until the BC diagnosis, mean (SD), days	NaN (NA)	621.70 (791.02)	NaN (NA)	498.51 (667.01)	NA
Charlson comorbidity index (CCI), mean (SD)	4.67 (2.49)	4.79 (2.45)	4.77 (2.51)	4.77 (2.47)	0.002
CCI group					0.014
0	142 (2.4)	87 (2.0)	86 (2.4)	79 (2.2)	
1-2	1059 (17.8)	722 (16.6)	608 (16.7)	617 (16.9)	
≥3	4756 (79.8)	3552 (81.4)	2953 (81.0)	2951 (80.9)	
Incomes, n (%)					0.015
Low	1231 (20.7)	936 (21.5)	798 (21.9)	775 (21.3)	
Middle	1499 (25.2)	995 (22.8)	850 (23.3)	860 (23.6)	
High	3227 (54.2)	2430 (55.7)	1999 (54.8)	2012 (55.2)	
BC diagnosis year, n (%)					0.125
2010-2013	1327 (22.3)	1122 (25.7)	821 (22.5)	1009 (27.7)	
2014-2016	2131 (35.8)	1519 (34.8)	1283 (35.2)	1254 (34.4)	
2017-2019	2499 (42.0)	1720 (39.4)	1543 (42.3)	1384 (37.9)	

© 2023 An MH et al. *JAMA Network Open*

Other cancers before BC diagnosis, n (%)	3332 (55.9)	2500 (57.3)	2141 (58.7)	2071 (56.8)	0.039
Alpha-blocker, n (%)	5957 (100.0)	4361 (100.0)	3647 (100.0)	3647 (100.0)	<0.001
Beta-3 agonist, n (%)	278 (4.7)	232 (5.3)	190 (5.2)	190 (5.2)	<0.001
Anticholinergics, n (%)	1556 (26.1)	1284 (29.4)	1031 (28.3)	1044 (28.6)	0.008
Anti-hypertensive agent, n (%)	3072 (51.6)	2522 (57.8)	2131 (58.4)	2072 (56.8)	0.033
Anti-atherosclerotic drug, n (%)	2346 (39.4)	1828 (41.9)	1543 (42.3)	1496 (41.0)	0.026
Male hormone, n (%)	11 (0.2)	9 (0.2)	6 (0.2)	8 (0.2)	0.013
Hypoglycemic agent, n (%)	1335 (22.4)	1062 (24.4)	884 (24.2)	893 (24.5)	0.006

SMD greater than 0.10 can be considered a sign of imbalance. To score the CCI, patients' previous history of myocardial infarction, congestive heart failure, peripheral vascular disease, cerebrovascular disease, dementia, chronic pulmonary disease, rheumatologic disease, peptic ulcer disease, mild liver disease, diabetes without chronic complication, diabetes with chronic complication, hemiplegia or paraplegia, renal disease, any malignancy including leukemia and lymphoma, moderate or severe liver disease, metastatic solid tumor, and acquired immune deficiency syndrome/human immunodeficiency virus were collected. BC, bladder cancer; PSM, propensity score matching; 5ARI, 5-alpha reductase inhibitor; AB, alpha blocker; SMD, standardized mean difference; SD, standard deviation.

© 2023 An MH et al. *JAMA Network Open*

eTable 3. Crude and adjusted hazard ratios of clinical outcomes associated with ≥ 2 filled prescriptions of 5ARIs in bladder cancer patients

All-cause mortality							
Prediagnostic prescription	No. of exposed	No. of cases	Person-year	Incidence rate (95% CI)†	Crude HR	Adjusted HR (95% CI)	Difference in RMST, days (95% CI)
AB only	5300	920	16275	56.53 (52.93-60.30)	Reference	Reference	Reference
5ARI (+AB)	5300	805	17470	46.08 (42.95-49.38)	0.82 (0.75-0.90)	0.83 (0.75-0.91)	92.6 (25.7-159.4)
Radical cystectomy							
Prediagnostic prescription	No. of exposed	No. of cases	Person-year	Incidence rate (95% CI)	Crude HR	Adjusted HR (95% CI)	Difference in RMST, days (95% CI)
AB only	5300	299	15282	19.57 (17.41-21.91)	Reference	Reference	Reference
5ARI (+AB)	5300	227	16737	13.56 (11.86-15.45)	0.73 (0.62-0.87)	0.74 (0.62-0.88)	68.0 (31.6-104.3)
Bladder instillation							
Prediagnostic prescription	No. of exposed	No. of cases	Person-year	Incidence rate (95% CI)	Crude HR	Adjusted HR (95% CI)	Difference in RMST, days (95% CI)
AB only	5300	1066	12455	85.59 (80.53-90.88)	Reference	Reference	Reference
5ARI (+AB)	5300	929	13985	66.43 (62.22-70.84)	0.84 (0.77-0.91)	0.84 (0.77-0.92)	88.1 (25.2-150.9)

The models were adjusted for age, smoking, BMI, CCI, and income level. To score the CCI, patients' previous history of myocardial infarction, congestive heart failure, peripheral vascular disease, cerebrovascular disease, dementia, chronic pulmonary disease, rheumatologic disease, peptic ulcer disease, mild liver disease, diabetes without chronic complication, diabetes with chronic complication, hemiplegia or paraplegia, renal disease, any malignancy including leukemia and lymphoma, moderate or severe liver disease, metastatic solid tumor, and acquired immune deficiency syndrome/human immunodeficiency virus were collected. RMST, Restricted mean survival time; HR, hazard ratio; BC, bladder cancer; 5ARI, 5-alpha reductase inhibitor; AB, alpha blocker; CI, confidence interval. † Per 1,000 person-years

© 2023 An MH et al. *JAMA Network Open*

eTable 4. Crude and adjusted hazard ratios of clinical outcomes associated with continuous user of 5ARIs in bladder cancer patients

All-cause mortality							
Prediagnostic prescription	No. of exposed	No. of cases	Person-year	Incidence rate (95% CI)†	Crude HR	Adjusted HR (95% CI)	Difference in RMST, days (95% CI)
AB only	3647	700	10986	63.72 (59.08-68.62)	Reference	Reference	Reference
5ARI (+AB)	3647	626	12423	50.39 (46.52-54.50)	0.80 (0.72-0.89)	0.82 (0.73-0.91)	136.0 (54.8-217.3)
Radical cystectomy							
Prediagnostic prescription	No. of exposed	No. of cases	Person-year	Incidence rate (95% CI)	Crude HR	Adjusted HR (95% CI)	Difference in RMST, days (95% CI)
AB only	3647	216	10253	21.07 (18.35-24.07)	Reference	Reference	Reference
5ARI (+AB)	3647	156	11958	13.05 (11.08-15.26)	0.69 (0.56-0.84)	0.69 (0.56-0.85)	85.0 (40.9-129.1)
Bladder instillation							
Prediagnostic prescription	No. of exposed	No. of cases	Person-year	Incidence rate (95% CI)	Crude HR	Adjusted HR (95% CI)	Difference in RMST, days (95% CI)
AB only	3647	727	8384	86.71 (80.52-93.25)	Reference	Reference	Reference
5ARI (+AB)	3647	652	9878	66.01 (61.04-71.27)	0.86 (0.77-0.95)	0.87 (0.78-0.96)	88.8 (14.6-163.0)

The models were adjusted for age, smoking, BMI, CCI, and income level. To score the CCI, patients' previous history of myocardial infarction, congestive heart failure, peripheral vascular disease, cerebrovascular disease, dementia, chronic pulmonary disease, rheumatologic disease, peptic ulcer disease, mild liver disease, diabetes without chronic complication, diabetes with chronic complication, hemiplegia or paraplegia, renal disease, any malignancy including leukemia and lymphoma, moderate or severe liver disease, metastatic solid tumor, and acquired immune deficiency syndrome/human immunodeficiency virus were collected. RMST, Restricted mean survival time; HR, hazard ratio; BC, bladder cancer; 5ARI, 5-alpha reductase inhibitor; AB, alpha blocker; CI, confidence interval. † Per 1,000 person-years

© 2023 An MH et al. *JAMA Network Open*

eTable 5. The association between total pre-diagnostic duration of 5ARI prescription and radical cystectomy in those with 365 days or more filled prescription of 5ARI

Prediagnostic prescription duration of 5ARI	Group	No. of exposed	No. of cases	Crude HR (95% CI)	Adjusted HR (95% CI)
**Total duration**					
1 to 2 years	AB only	729	55	Reference	Reference
1 to 2 years	5ARI (+AB)	1187	68	0.74 (0.52-1.06)	0.79 (0.55-1.13)
2 years and more	AB only	3697	201	Reference	Reference
2 years and more	5ARI (+AB)	3239	122	0.78 (0.63-0.96)	0.77 (0.62-0.95)

Models were adjusted for age, smoking, BMI, CCI, and income level.

© 2023 An MH et al. *JAMA Network Open*

eTable 6. The association between 5ARI prescriptions (≥2 filled) and progression in bladder cancer patients based on different exposure lag periods prior to bladder cancer diagnosis

	**Proportional hazards ratio (HR)†**		**Restricted mean survival time**	
	Unadjusted HR (95% CI)	Adjusted HR* (95% CI)	Difference in RMST (95% CI)	P value
**Lag: 12 months (main)**				
All-cause mortality	0.82 (0.75-0.90)	0.83 (0.75-0.91)	92.6 (25.8–159.4)	0.007
Radical cystectomy	0.73 (0.62–0.87)	0.74 (0.62–0.88)	68.0 (31.7–104.2)	<0.001
Bladder instillation	0.84 (0.77–0.91)	0.84 (0.77–0.92)	88.1 (25.2–151.0)	0.006
**Lag: 18 months**				
All-cause mortality	0.81 (0.73–0.90)	0.81 (0.73–0.90)	97.6 (24.9–170.3)	0.03
Radical cystectomy	0.72 (0.60–0.87)	0.73 (0.61–0.88)	73.0 (34.4–111.6)	0.005
Bladder instillation	0.83 (0.75–0.91)	0.83 (0.76–0.92)	93.9 (30.6–157.2)	0.002
**Lag: 24 months**				
All-cause mortality	0.81 (0.72–0.90)	0.81 (0.73–0.91)	89.6 (10.2–169.0)	0.03
Radical cystectomy	0.72 (0.59–0.88)	0.73 (0.60–0.89)	57.3 (17.5–97.1)	0.005
Bladder instillation	0.84 (0.76–0.93)	0.85 (0.77–0.94)	81.7 (14.5–148.9)	0.002

†Cox proportional hazards regression in matched cohort. *The models were adjusted for age, smoking, BMI, CCI, and income level. To score the CCI, patients' previous history of myocardial infarction, congestive heart failure, peripheral vascular disease, cerebrovascular disease, dementia, chronic pulmonary disease, rheumatologic disease, peptic ulcer disease, mild liver disease, diabetes without chronic complication, diabetes with chronic complication, hemiplegia or paraplegia, renal disease, any malignancy including leukemia and lymphoma, moderate or severe liver disease, metastatic solid tumor, and acquired immune deficiency syndrome/human immunodeficiency virus were collected. RMST, restricted mean survival time; HR, hazard ratio; 5ARI, 5-alpha reductase inhibitor; AB, alpha blocker.

© 2023 An MH et al. *JAMA Network Open*

eTable 7. Sensitivity analysis for RMST using diverse truncated time point for the association between 5ARI prescriptions and bladder instillation (365 days or more filled prescriptions)

Truncated time(year)	Difference in RMST (95% CI)	P Value
At 6 years	56.5 (10.4–102.6)	0.0167
At 7 years	64.4 (9.1–119.7)	0.0223
At 8 years	69.8 (5.3–134.3)	0.034
At 9 years	73.3 (-1.0–147.6)	0.0533
At 10 years	64.1 (-23.5–151.7)	0.15

A All users

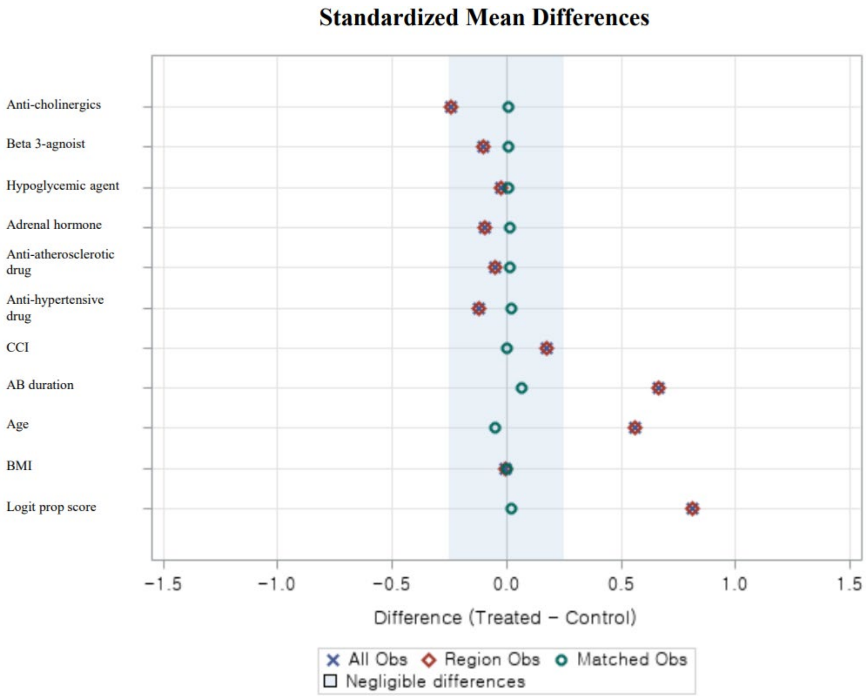

B Continuous users

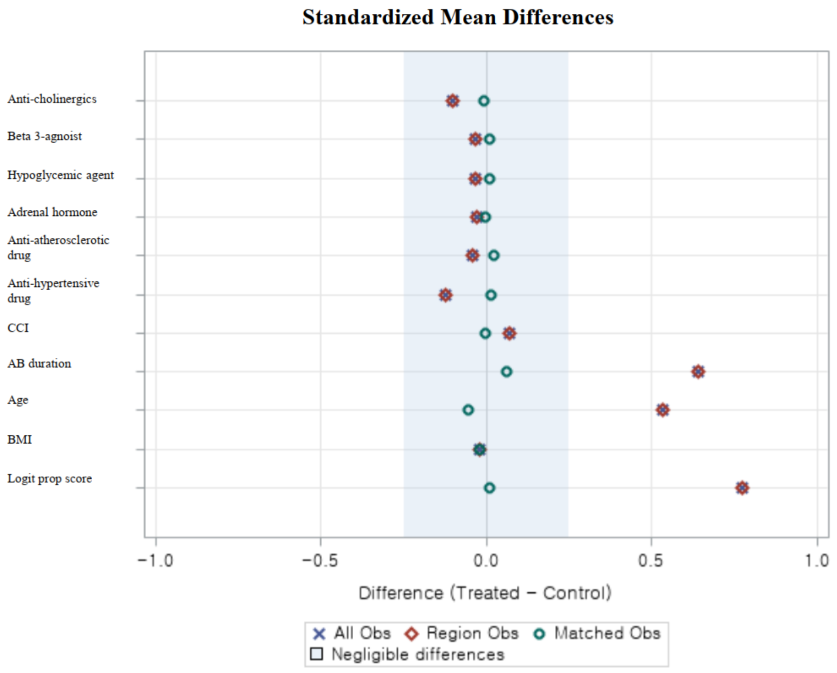

eFigure 1. Covariate balance between arms before and after propensity score matching (PSM) for (A) all users and (B) continuous users

Red dots represent before PSM and green dots represent after PSM. To score the Charlson comorbidity index (CCI), patients' previous history of myocardial infarction, congestive heart failure, peripheral

© 2023 An MH et al. *JAMA Network Open*

vascular disease, cerebrovascular disease, dementia, chronic pulmonary disease, rheumatologic disease, peptic ulcer disease, mild liver disease, diabetes without chronic complication, diabetes with chronic complication, hemiplegia or paraplegia, renal disease, any malignancy including leukemia and lymphoma, moderate or severe liver disease, metastatic solid tumor, and acquired immune deficiency syndrome/human immunodeficiency virus were collected. AB, alpha blocker; BMI, body mass index.

© 2023 An MH et al. *JAMA Network Open*

A All users – before PSM

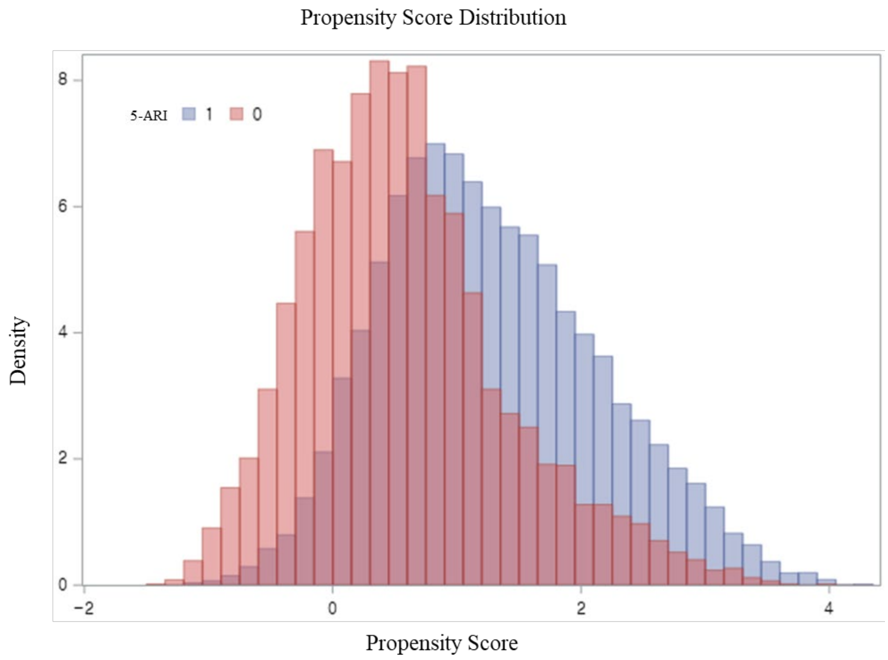

B All users – after PSM

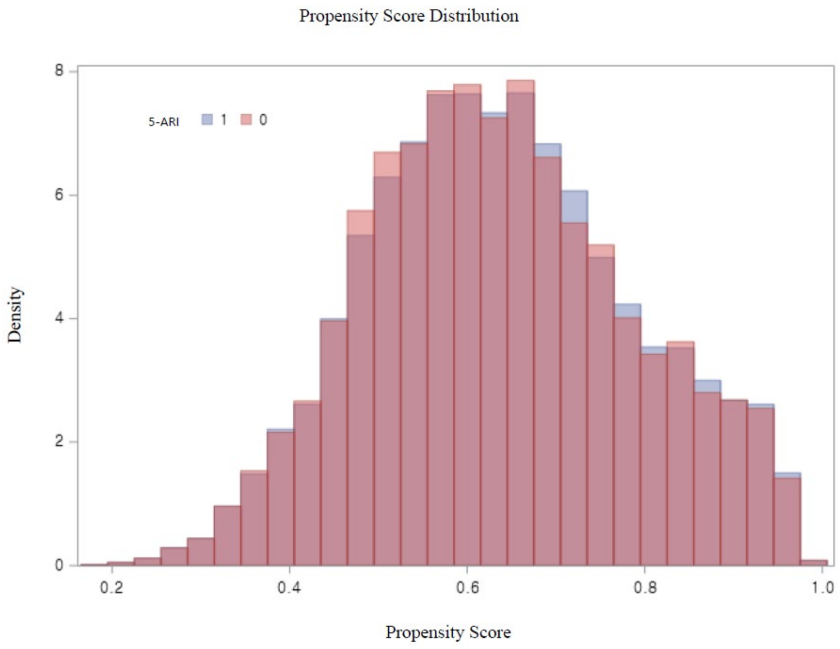

© 2023 An MH et al. *JAMA Network Open*

C Continuous users – before PSM

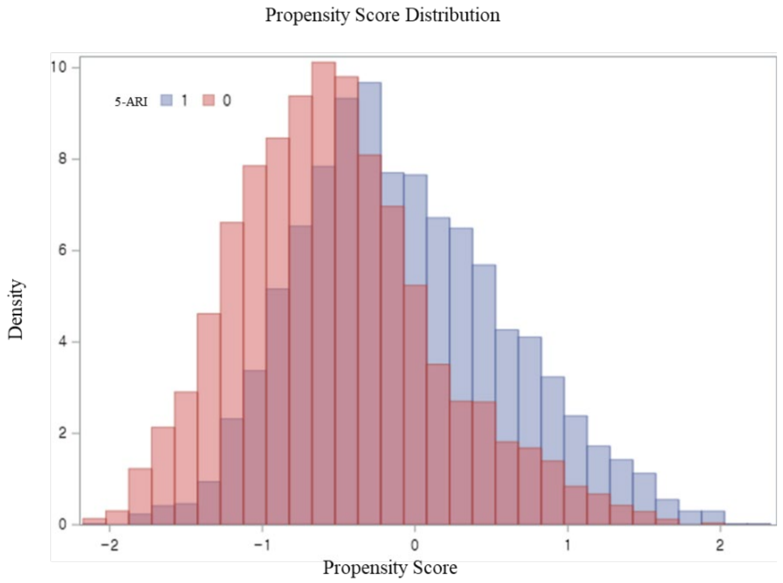

D Continuous users – after PSM

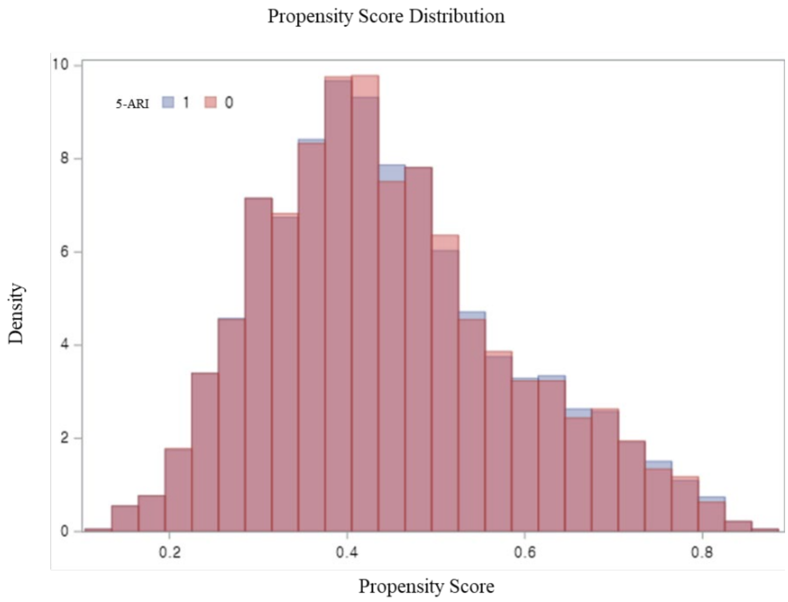

eFigure 2. Propensity score distribution of arms after matching for all users (A,B) and continuous users (C,D)

© 2023 An MH et al. *JAMA Network Open*

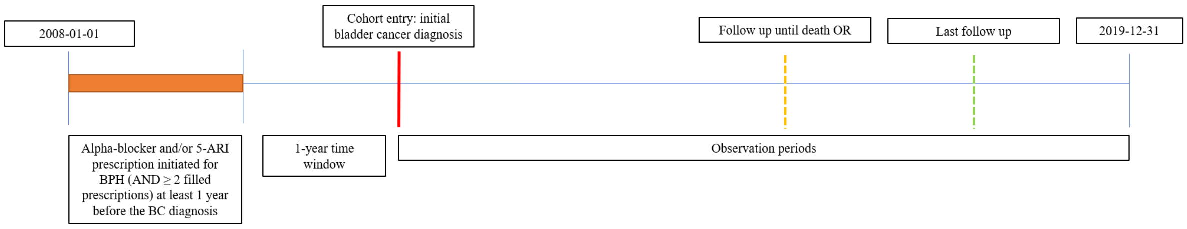

eFigure 3. Diagram of cohort construction

© 2023 An MH et al. *JAMA Network Open*

A Adjusted cox proportional model for all-cause mortality (all users)

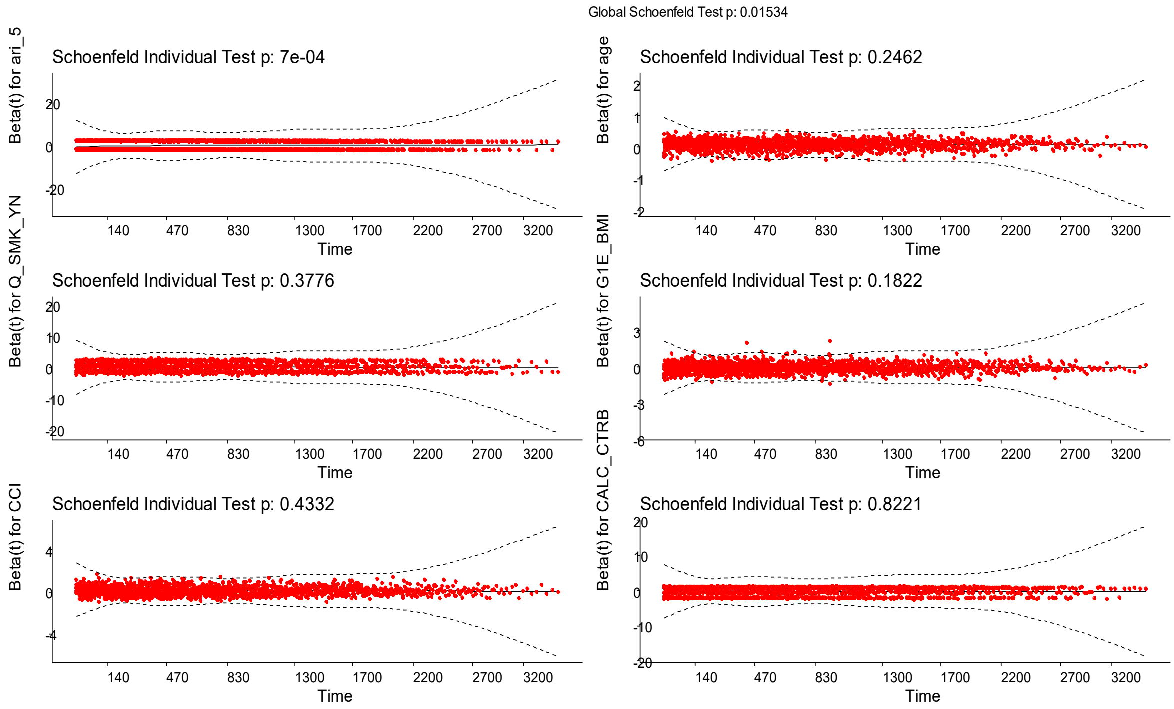

B Adjusted cox proportional model for all-cause mortality (continuous users)

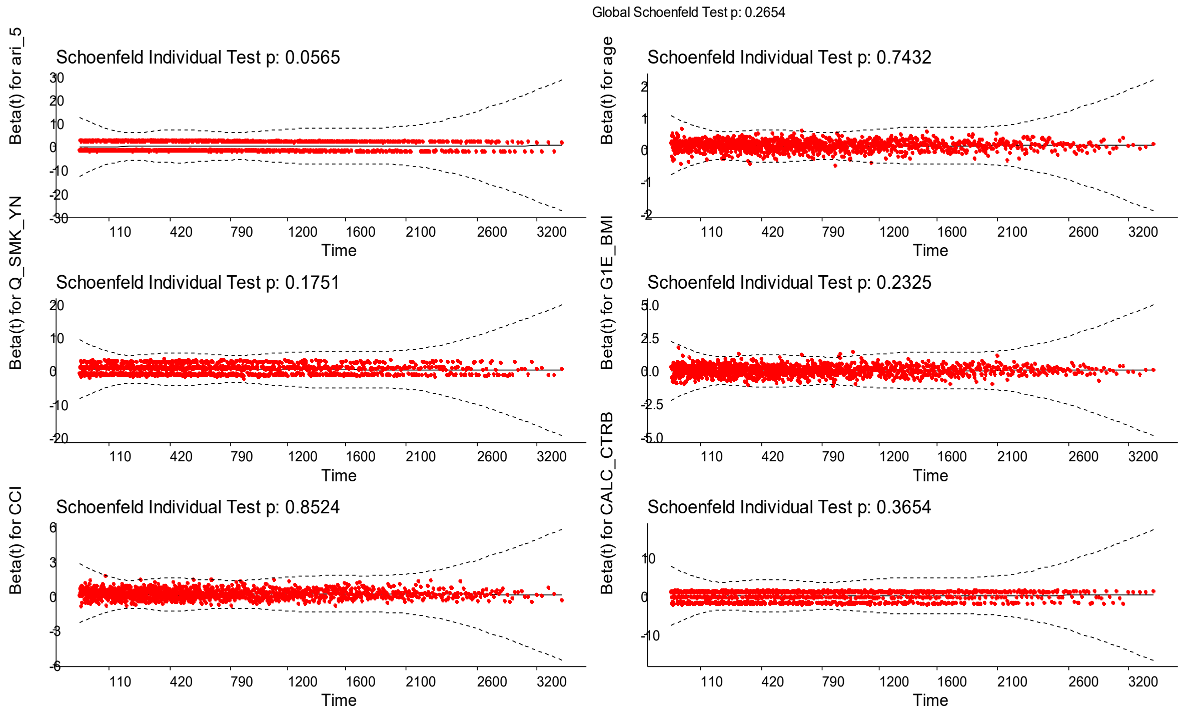

© 2023 An MH et al. *JAMA Network Open*

C Adjusted cox proportional model for bladder instillation (all users)

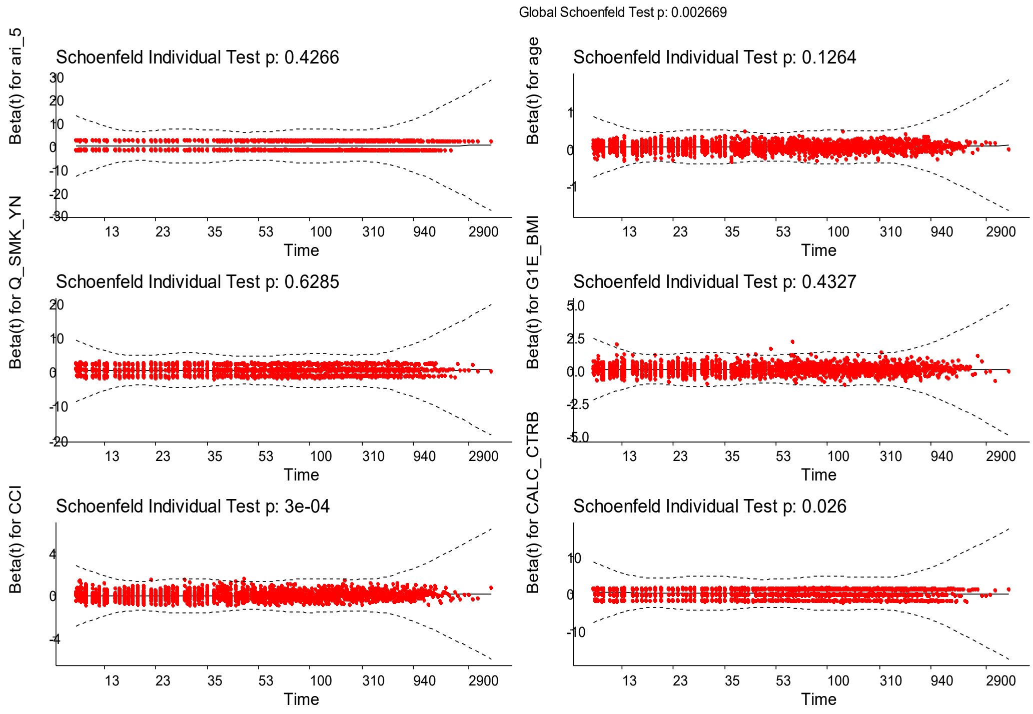

D Adjusted cox proportional model for bladder instillation (continuous users)

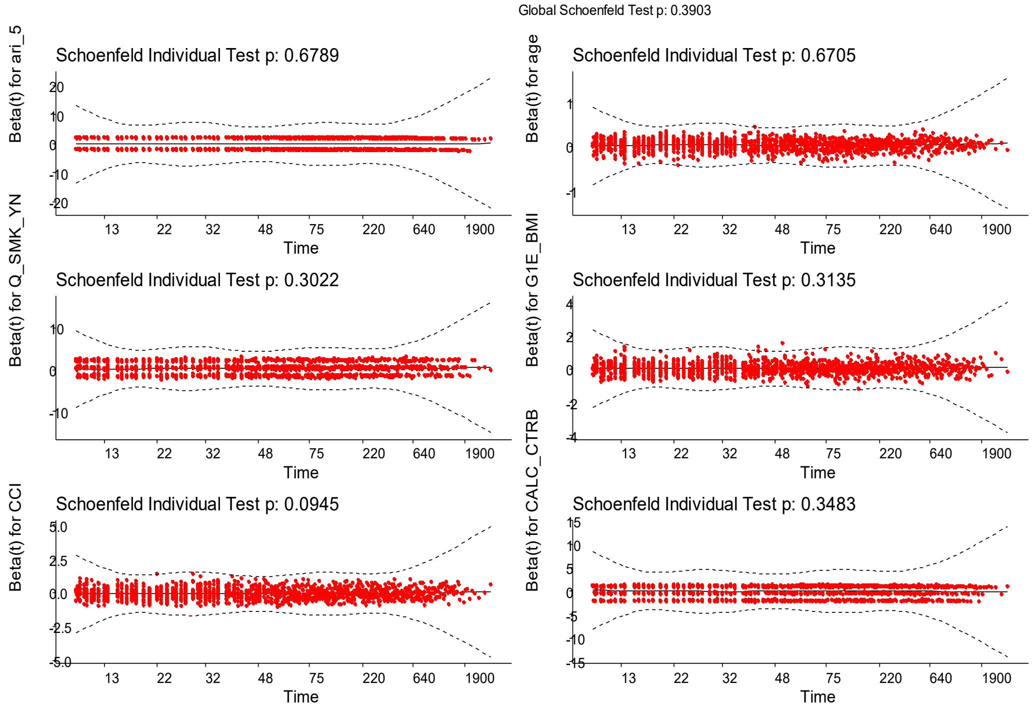

© 2023 An MH et al. *JAMA Network Open*

E Adjusted cox proportional model for radical cystectomy (all users)

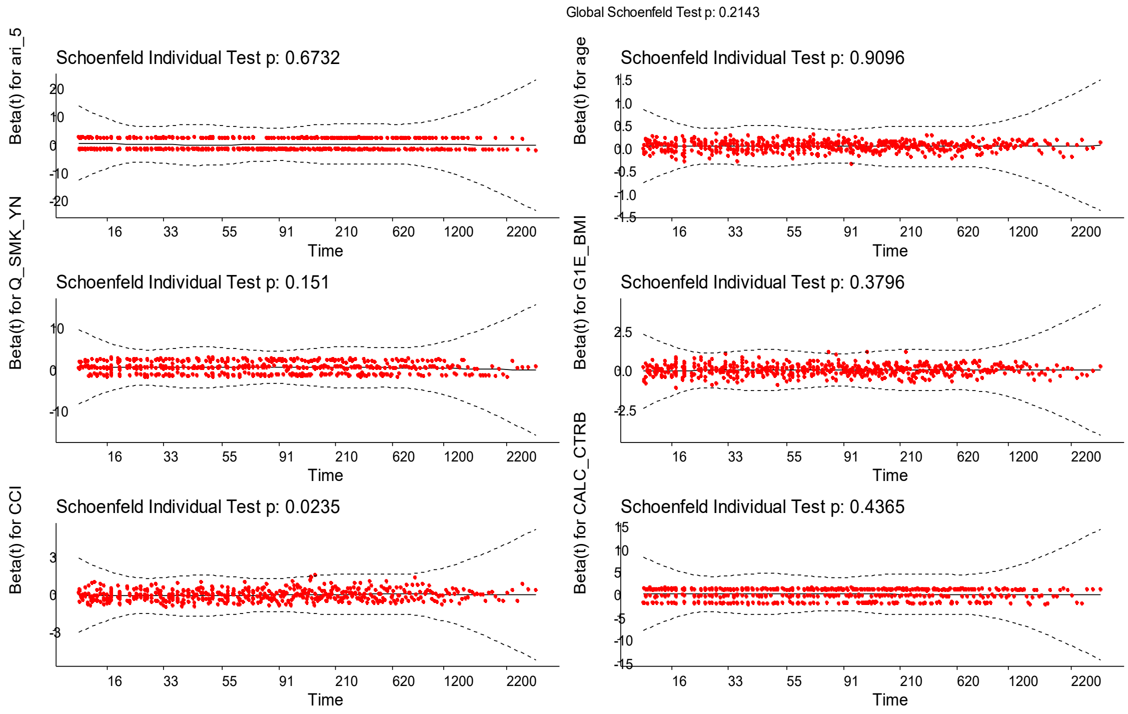

F Adjusted cox proportional model for radical cystectomy (continuous users)

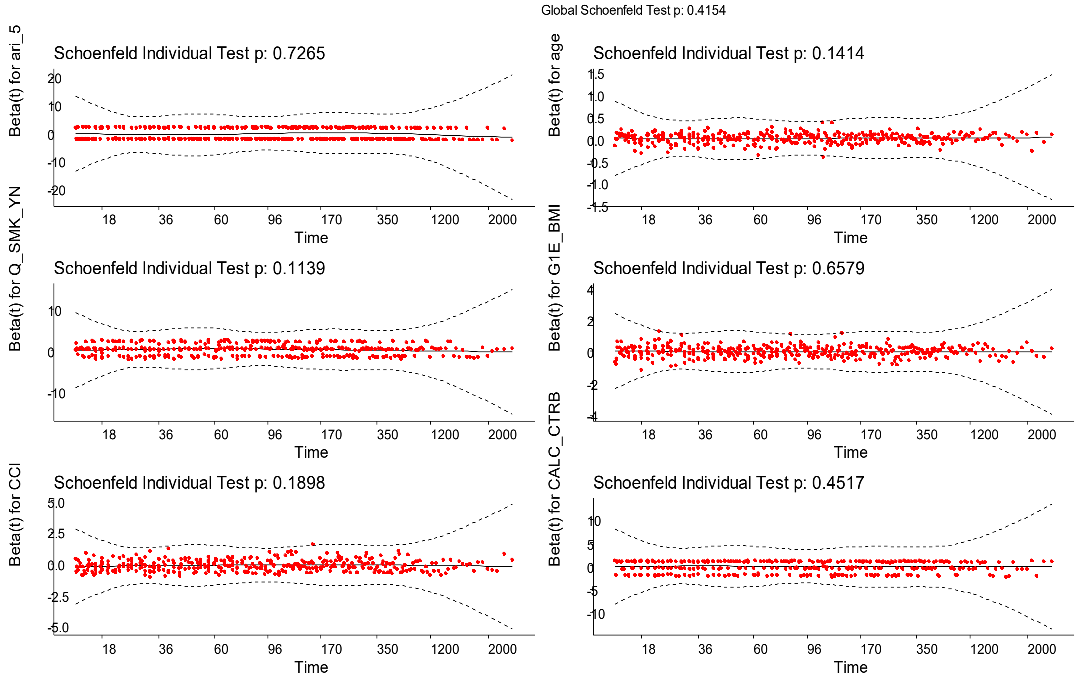

eFigure 4. Diagnostics for the proportional hazards assumption of the Cox proportional hazards model using Schoenfeld residuals

© 2023 An MH et al. *JAMA Network Open*

A All-cause mortality (all users)

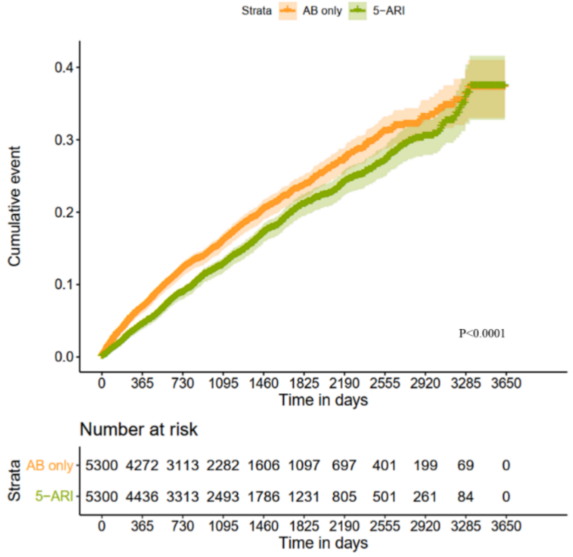

B Bladder instillation (all users)

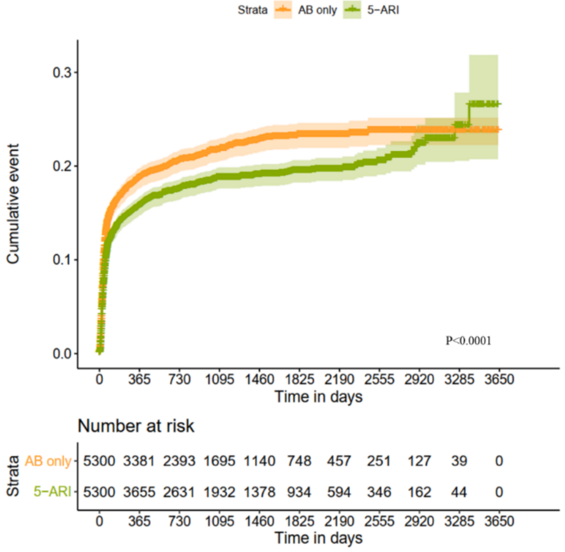

C Radical cystectomy (all users)

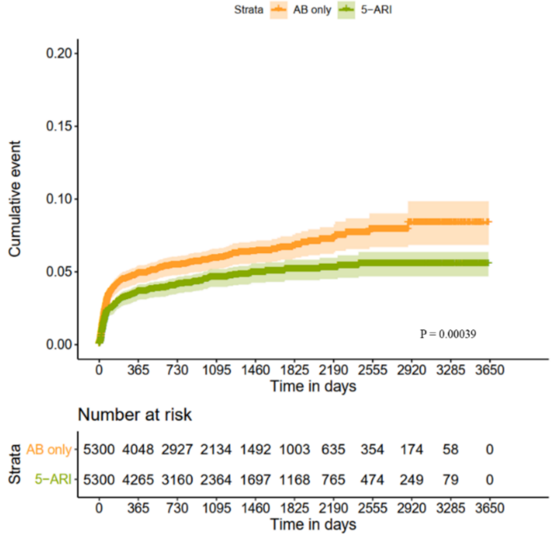

D All-cause mortality (continuous users)

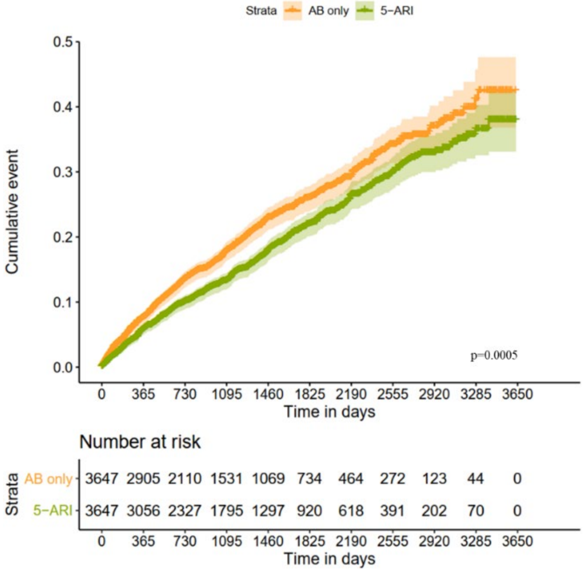

E Bladder instillation (continuous users)

F Radical cystectomy (continuous users)

© 2023 An MH et al. *JAMA Network Open*

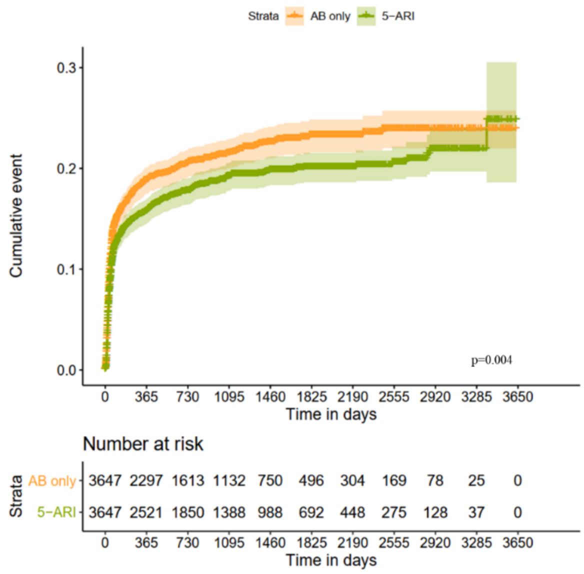

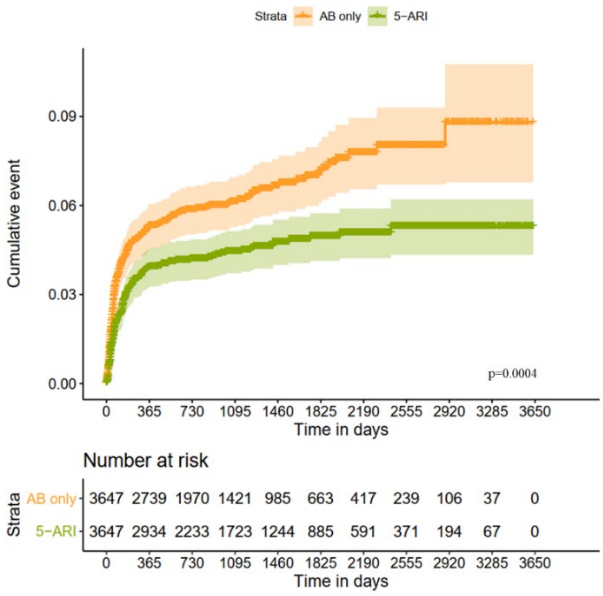

eFigure 5. Cumulative incidence plot of clinical outcomes for all (A,B,C) and continuous 5ARI users (D,E,F)

© 2023 An MH et al. *JAMA Network Open*
