# Supplementary material for: Association of 5α-Reductase Inhibitor Prescription With Bladder Cancer Progression in Males in South Korea
Source: JAMA Netw Open. 2023 May 16;6(5):e2313667. doi: 10.1001/jamanetworkopen.2023.13667 (PMC10189569; doi:10.1001/jamanetworkopen.2023.13667)
Supplement: Supplement 2. — Data Sharing Statement [file jamanetwopen-e2313667-s002.pdf]

Data Sharing Statement

An. Association of 5α-Reductase Inhibitor Prescription With Bladder Cancer Progression in Males in South Korea. *JAMA Netw Open*. Published May 16, 2023. doi:10.1001/jamanetworkopen.2023.13667

Data
Data available: No
